# The production of monodisperse explosive particles with piezo-electric inkjet printing technology

**DOI:** 10.1063/1.4938486

**Published:** 2015-12

**Authors:** M. E. Staymates, R. Fletcher, M. Verkouteren, J. L. Staymates, G. Gillen

**Affiliations:** The National Institute of Standards and Technology, Gaithersburg, Maryland 20899, USA

## Abstract

We have developed a method to produce discrete microparticles from compounds dissolved in nonpolar or polar solvents using drop-on-demand inkjet printer technology. A piezoelectric inkjet printhead located atop a drying tube produces precise droplets containing defined quantities of analyte. Droplets solidify into microparticles with known composition and size as they traverse down the drying tube. Because this is a drop-on-demand printing process, a known number of droplets are produced providing quantitative particle delivery to a variety of substrates. Particular emphasis is placed on the development and characterization of the drying tube in this work. The drying tube was modeled using computational fluid dynamics and experimentally evaluated using laser-based flow visualization techniques. A notable design feature of the drying tube is the ability to push heated air through the tube rather than the need to pull air from the exit. This provides the ability to place a known number of well-defined particles onto almost any substrate of interest, rather than having to collect particles onto a filter first and then transfer them to another surface. Several types of particles have been produced by this system, examples of which are pure particles of cyclotrimethylenetrinitramine ranging from 10 μm to 30 μm in diameter, and ammonium nitrate particles of 40 μm diameter. The final particle size is directly related to the solute concentration of the printing solution and the size of the initial jetted droplet.

## I. INTRODUCTION

Trace explosives detection is a rapidly evolving screening technology based on the analysis of the chemical signatures from trace residues of explosives. These residues are typically in the form of small solid particles that are a few to tens of micrometers in diameter.^1^ The residues result from the transfer of small particles from a bulk explosive material onto a person or their belongings. This transfer can result from a person carrying explosives, manipulation or manufacturing of explosives, or being contaminated by proximity in such an environment. Explosive trace detectors (ETDs) are now widely deployed to sample and screen for these residues.

Two approaches are used for collecting trace explosive residues: aerodynamic sampling and swipe-based sampling. In aerodynamic sampling systems, an object is interrogated by a series of pulsed air-jets, air-blades, and/or fans that seek to liberate contraband particles from the surface, and then aerodynamically transport them to a collection device.^2^ In swipe sampling,^3^ particles are collected by drawing a swab of cloth across a suspect surface. In each approach, collected particles are then thermally desorbed and analyzed by a trace chemical detector, typically an ion mobility spectrometer (IMS).^4^

This work is motivated by the development and application of trace chemical standards/test materials for sampling effciency measurements and instrument calibration and optimization. Our interest here is in developing trace explosive particulate test materials to support the optimization of currently deployed detection systems as well as accelerate the development of next-generation trace sampling technology. These test materials must be well characterized in terms of size, shape, and chemical composition, and should simulate trace explosive contamination in the form of small discrete particles with characteristic diameters in the 5 μm –30 μm range.^1^ Previously, high explosives have been successfully incorporated into monodisperse polymer microspheres using a piezo-electric inkjet printing method^5^ and by using a precision particle fabrication technique.^6^ However, there is increased interest in making pure explosive particles to facilitate evaluation or calibration of next-generation trace detection technologies.

Applications and fields-of-study for monodisperse droplets/particles are as wide-ranging as the techniques used to produce them. These fields relate to chemical engineering,^7^ electronics and solar cells,^8^ 3D printing and additive manufacturing,^9^ food and agriculture,^10,11^ powder metallurgy,^12^ ceramics,^13^ and many others. In this work, we present a method for producing monodisperse microparticles by piezoelectric inkjet printing combined with a unique drying tube. A recent publication describes in some detail the technology and state of the art along with showing the emerging current interest in inkjet printing to make particles.^14^

Commercial inkjet printheads that are largely based on “bubble-jet” technology have been shown to be effective in producing microparticles from polar solvents and water soluble materials.^15–17^ Piezo-electric inkjet printers, which eject droplets based on the electromechanical deformation of a piezo-electric transducer, expand the particle domain by enabling microparticle creation from not only water soluble compounds, but those compounds that are largely soluble in organic solvents. As an example, the vibrating orifice particle generator is a continuous piezoelectric inkjet dispenser and can produce large numbers of particles (particle generation frequency is on the order of the piezo frequency) from a wide range of solvents.^18,19^ On the other hand, drop-on-demand piezoelectric inkjet printers deliver a well-defined and more controlled number of particles per unit time.^20^ The drop-on-demand method provides a highly precise and repeatable means for depositing picoliter volumes of solution onto surfaces,^21^ and has been used to prepare highly accurate and reproducible standard test materials for performance verification of a variety of ETDs.^22^

One particular advantage of drop-on-demand piezoelectric inkjet printing is the ability to precisely control the particle diameter to fabricate micrometer sized particles by changing the initial printing solution concentration. By controlling the diameter, we can tailor these standard test particles to mimic the true threat that exists from real-world trace explosive contamination. Our interest here is in producing a known number of test particles with analytically known amounts of compound, a specified monodisperse particle size distribution, and the ability to deliver these particles onto a diverse range of substrates that represent surfaces that are relevant to security checkpoints. Building upon a system initially designed by the U.S. Army,^17^ a new drying tube has been designed, fabricated, and tested to produce monodisperse particles made of explosive compounds. The new drying tube and optimized control of the inkjet printer provide an improved particle generator for our specific homeland security application.

## II. MATERIALS AND METHODS

### A. System design and optimization

The Particle Jet is a device that uses a Microfab piezoelectric inkjet device to deliver a known number of uniform liquid droplets into a vertical, heated drying tube. The solution that is being dispensed consists of an explosive material dissolved in an aqueous or organic solvent. This solvent evaporates as the liquid droplets travel through the drying tube, producing solid spherical particles made of pure explosives. Particles are then collected on a substrate located at the exit of the drying tube.

The piezo-electric inkjet nozzle requires precise control of fluid backpressure in order to operate effectively. Since slight perturbations in pressure can cause dispensing instability and satellite production, a dynamic pressure control system was developed to be used with the inkjet that regulates the backpressure with great accuracy.^20,21^ Basic lab pressure and lab vacuum are also required to operate the inkjet nozzle.

**FIG. 1:**
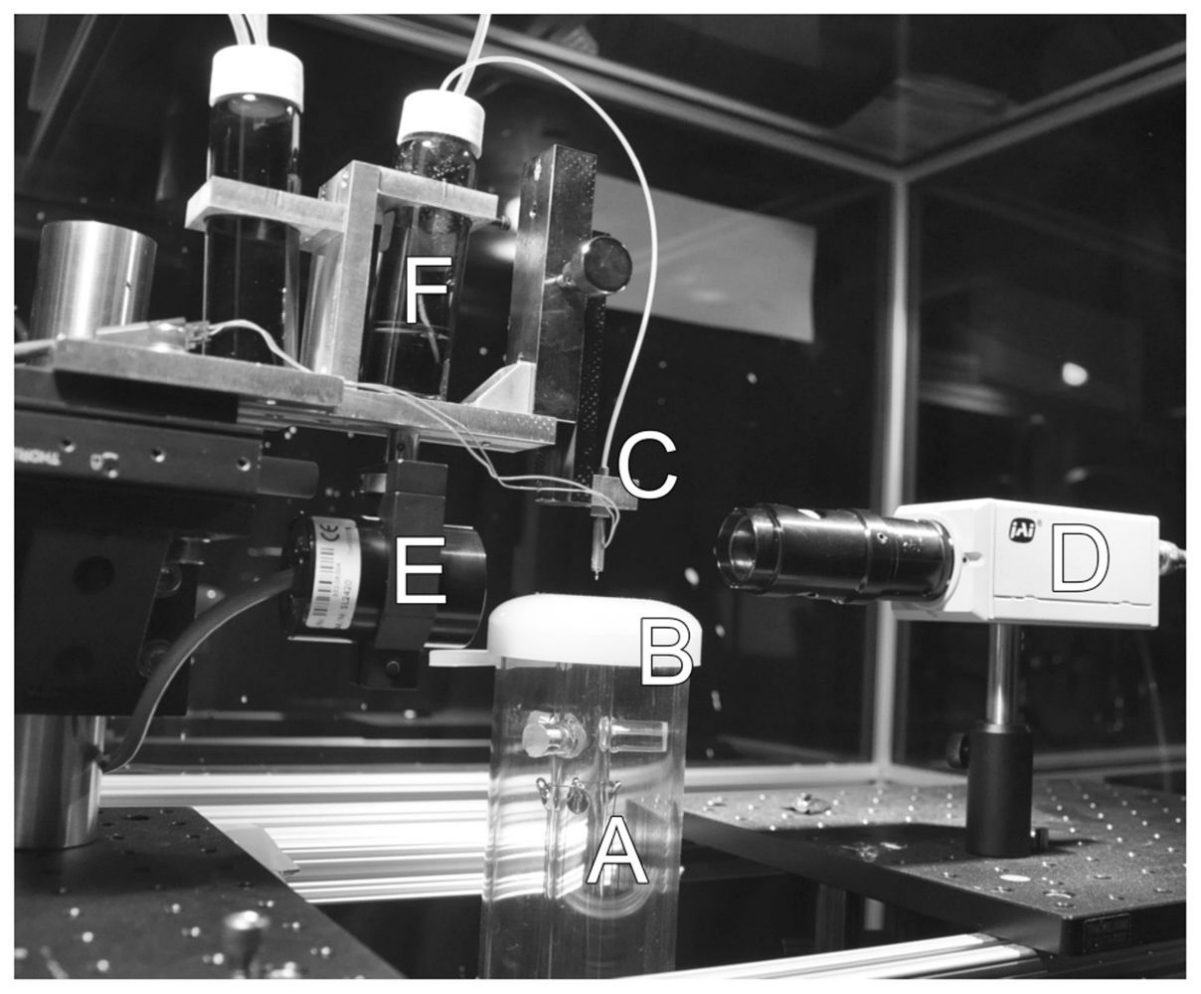
An image of the upper section of the particle jet. The top of the drying tube is labeled (a), (b) is the flow-focusing cap, (c) is the piezo electric inkjet nozzle, (d) is the digital camera used to visualize the droplets, (e) is the strobe light used to illuminate the droplets, and (f) is the ink reservoir.

The inkjet device is tuned prior to a production run to determine the proper conditions for stable droplet production. Tuning is facilitated by adjusting the voltage waveform that is applied to the piezo element and visualizing the droplet formation, via stroboscopic visualization,^23^ until a stable droplet is produced. Once the printhead is operating properly and producing consistent droplets, it is positioned over the drying tube and then particle production can begin. An image of the upper portion of the system is shown in Figure 1.

**FIG. 2:**
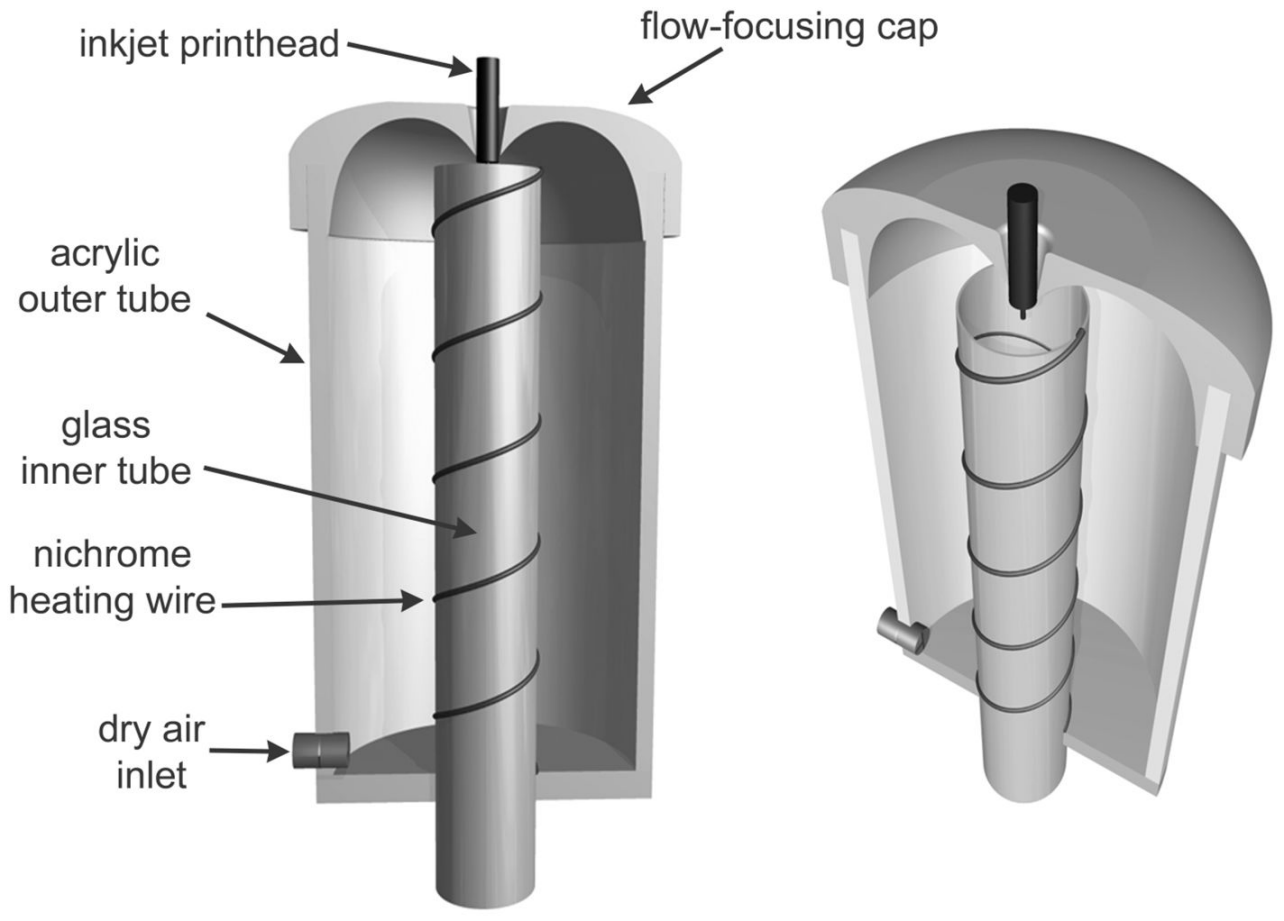
Schematic diagram of the second-generation drying tube consisting of the flow-focusing cap, heating wire, and inner and outer tubes. Half of the cap and outer tube have been removed to visualize the interior components. The image on the right shows a 3D perspective view that illustrates how the inkjet printhead is positioned into the central tube and how the nichrome wire wraps around the central tube. This figure serves as an illustration and is not drawn to scale.

The drying tube is designed for controlled single or multi-particle delivery. The drying tube is 760 mm long and consists of an acrylic outer tube with an outer diameter of 64 mm and 1.5 mm wall thickness. An inner glass tube of 22 mm outer diameter and 1 mm wall thickness is mounted concentrically to the outer glass tube. A flow-focusing cap is located at the top of the outer wall assembly and is crucial to the effective flow control in this drying tube, as it creates a flow pattern within this domain that vectors droplets downward through the tube without the need for suction at the bottom (see Figure 2). This cap was fabricated on an Objet 30 Pro 3D printing system (Stratasys, Inc., Eden Prairie, MN). The inkjet printhead is positioned in the center of the flow-focusing cap, but sits down inside the central tube. Nichrome heating wire supplies heat to the inner glass tube to facilitate rapid and complete evaporation of solvent during droplet transport through the system. The particles must be completely dry (solvent free) when they reach the end of the tube and impact onto a substrate of interest.

Regulated dry air is delivered into the acrylic tube through a mass flow controller (Omega Inc., Stamford, CT). As air enters the chamber and travels upwards, it is heated by the nichrome wire heating elements that wrap the inner glass tube. The heated air eventually reaches the upper cap and is then focused radially inwards towards the inkjet nozzle. Then, the flow is vectored downwards through the inner tube and ultimately leaves at the bottom through the exit orifice. The flow rate in this system can be adjusted from zero to 0.5 liters per minute (LPM).

**FIG. 3:**
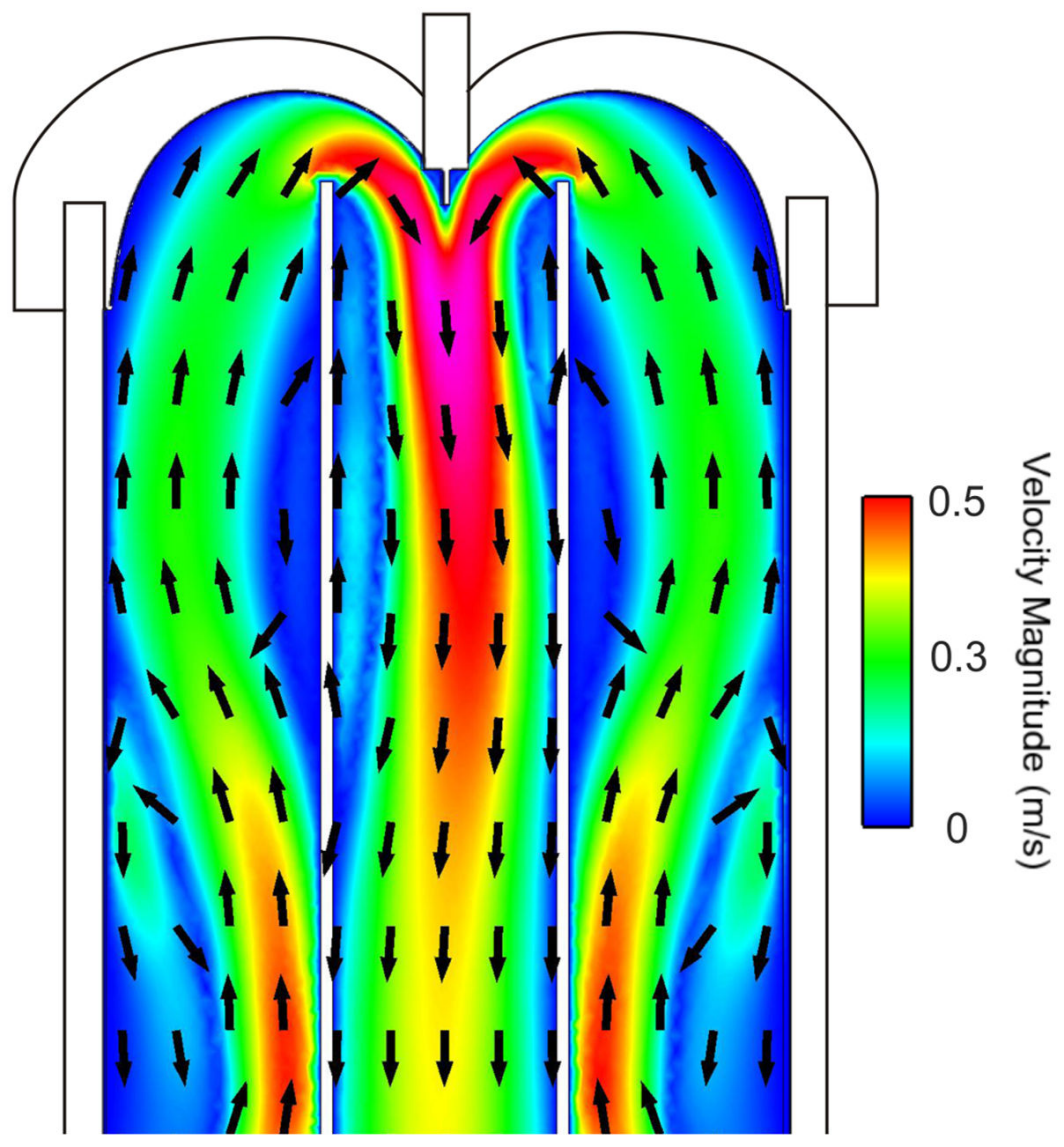
CFD results of the velocity field illustrated as color contours and velocity vectors. The velocity range is shown in the scale to the right. Only the upper section of the model is shown here for simplicity.

**FIG. 4:**
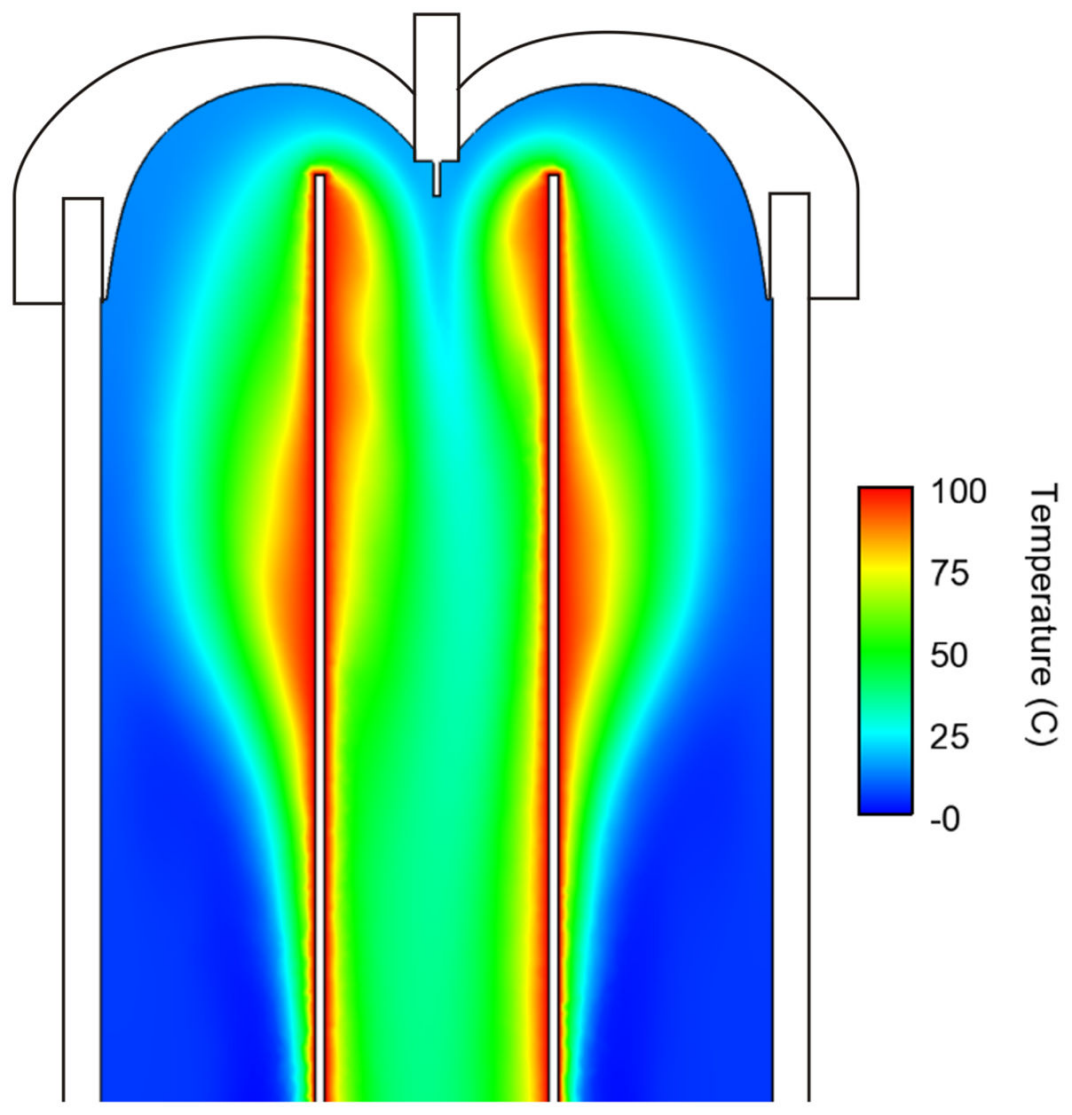
CFD results of the temperature field found during normal operation with the wall temperature set to 100 °C. Only the upper section of the model is shown here for simplicity.

The internal flow dynamics of the heated drying tube were modeled using computational fluid dynamics (CFD) modeling (CFD-ACE software, ESI group, San Diego, CA). This technique has been particularly useful in studying the internal fluid mechanics of ETDs,^24^ and is now being employed to assist in the design of standards to test these ETDs. Simulations were performed at steady state, laminar conditions. An example of the velocity flow field inside the drying tube operating at an inlet flow rate of 0.5 l/min and a temperature of 100 °C is shown in Figure 3. The temperature field is shown in Figure 4. This model is 2-dimensional because of the axisymmetric nature of the domain.

These results show that the velocity field within the drying tube is directed upwards along the outer tube and downwards within the inner glass tube. Without the presence of the flow-focusing cap, buoyancy from the heated walls would drive an upward air flow within the inner glass tube and carry droplets/particles vertically away from the system. This is precisely why the flow-focusing cap was designed; to counter the upward buoyant forces and to drive droplets and particles downwards through the inner glass tube without the need for vacuum suction at the bottom of the tube.

These CFD results also show that the fluid near the walls of the central tube is heated to approximately 100 °C with some lateral variation. Thermal fields near the top in the center of the domain are in the range of 35 °C–50 °C. The temperature profile along a cross-section of the inner tube becomes approximately 50 °C and fairly uniform at about 5 or 6 tube diameters downstream from the inkjet printhead.

**FIG. 5:**
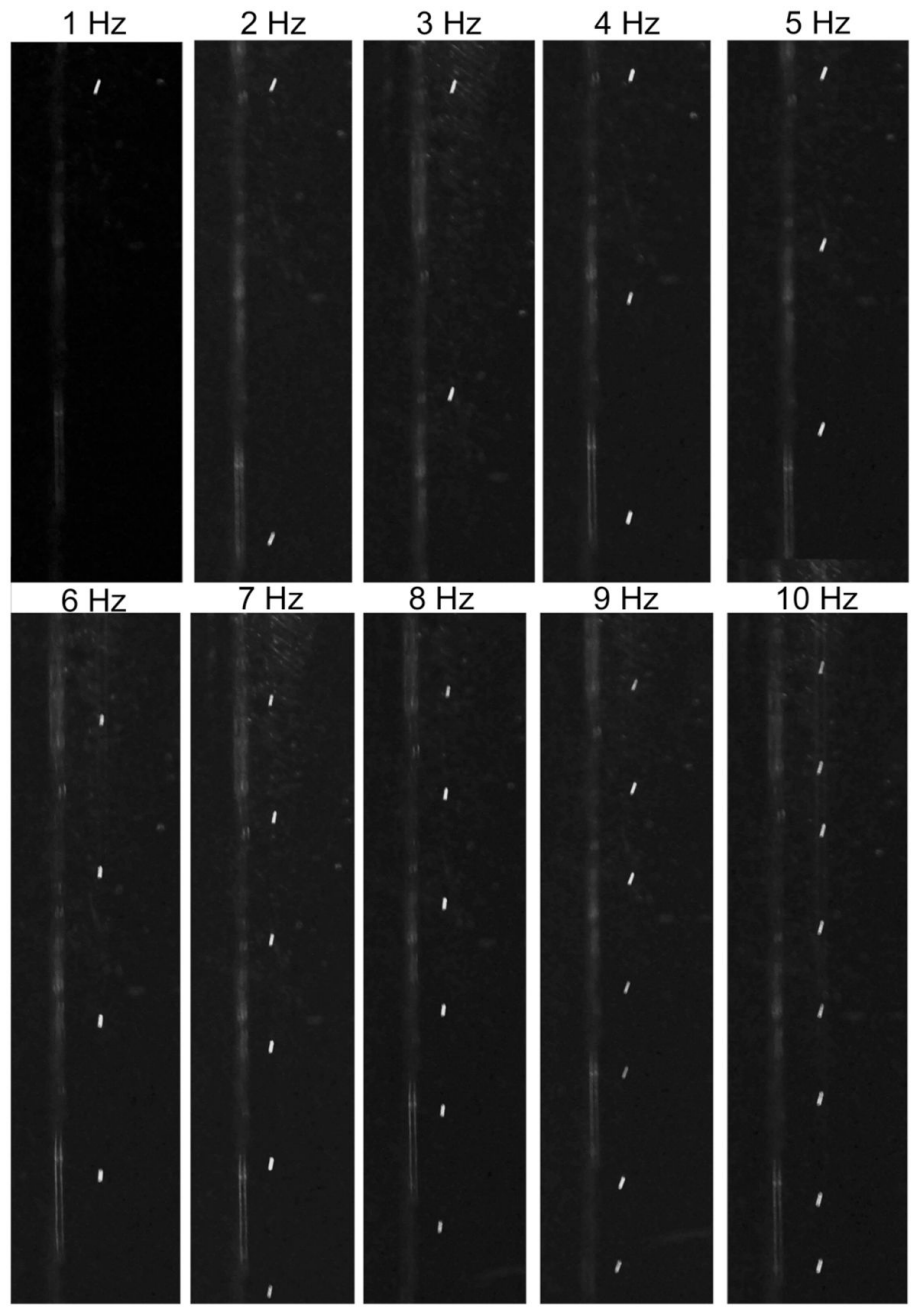
Flow visualization of droplet spacing as a function of jetting frequency. These images were taken close to the top of the drying tube, enabling the camera to capture the individual droplets. The droplets appear non-spherical due to the extended camera exposure time needed to capture the images.

Fluid flow visualization experiments were performed to study the behavior of the droplets as they transit through the drying tube. In these experiments, a laser light sheet bisects the long axis of the tube from the bottom to the top. Droplets delivered into the tube from the inkjet nozzle are illuminated by the laser light as they travel downwards. A digital camera is used to capture the droplets in flight. To demonstrate the influence of jetting frequency on droplet behavior and spacing, images of droplets were collected at varying jetting frequencies ranging from 1 Hz to 10 Hz. A collage of these images is given in Figure 5. The flow rate through the tube was 0.5 l/min and pure water was used as the jetting solvent.

As expected, the spacing between droplets becomes smaller as the jetting frequency increases. With no wall heat applied, the droplets follow an almost-vertical trajectory. This suggests that a mechanical x-y translation stage may be used at the bottom of the drying tube to provide a systematic or custom particle deposition pattern. The droplet spacing is on the order of tens to hundreds of droplet diameters. As droplet spacing becomes increasingly smaller, droplet-droplet interactions begin to affect the resulting particle distribution. Droplet coalescence^25^ and an increase of the solvent vapor pressure around the evaporating droplets^26^ can affect the evaporation characteristics of the droplets in flight. To this end, we have found that particle production with this system is at optimum performance levels when the droplet ejection frequency is 10 Hz or less. Frequencies above 10 Hz result in particle aggregates, incomplete particle drying, and coalescence of droplets in flight.

### B. Production of cyclotrimethylenetrinitramine (RDX) particles

Determination of an optimized analyte-solvent combination can be an especially challenging process, mainly due to the compatibility of the solvent with the piezoelectric inkjet nozzle. There is a narrow window of acceptable fluid viscosity and vapor pressure that the solvent must possess. Low viscosities usually lead to satellite formation and residual pressure wave interaction between droplets. In contrast, high viscosities cause energy dissipation and hinder the formation of droplets.^27^ The evaporation rate of the solvent must be sufficiently high to produce dry particles at the exit of the drying tube. If evaporation occurs too rapidly, the droplet formation tends to be unstable and the analyte may crystallize within the inkjet nozzle and grow into a blockage. To prepare relatively large (>10 μm) RDX particles, the analyte must be highly soluble in the solvent. We found that dimethyl formamide (DMF) solvent is a suitable solvent for printing RDX particles, as RDX is highly soluble in DMF. RDX crystals were dissolved in DMF (Sigma Aldrich) to create solution concentrations of approximately 150 mg/ml, 100 mg/ml, 50 mg/ml, 25 mg/ml, and 5 mg/ml. For the resulting particles shown in this work, the flow rate through the drying tube was set at 0.5 LPM and the temperature of the drying tube was set to 50 °C.

### C. Production of ammonium nitrate (AN) particles

Ammonium nitrate is highly soluble in water, allowing the preparation of an aqueous solution concentration of approximately 300 mg/ml. Bulk AN powder was placed in a desiccator overnight to remove as much moisture as possible from the deliquescent material and to enable accurate weighing using an analytical balance. The flow rate through the drying tube in this production run was set at 0.3 LPM and the temperature of the drying tube was set to 100 °C, a larger temperature setpoint than the RDX production run, to facilitate enhanced evaporation of water from the droplets in flight.

### D. Data collection and imaging

To maintain a stable droplet ejection, printing parameters were fine-tuned so that the droplet had no satellites and remained stable. Once the droplet and parameters were optimized, clean silicon wafers were placed on the filter at the bottom of the drying tube and the nozzle was positioned at the top of the drying tube and lowered into the flow-focusing cap. For each RDX solution concentration, 1000 drops were dispensed at a frequency of 10 Hz. AN microparticles were dispensed at 5 Hz. Images of the resulting microparticles were captured using a scanning electron microscope (SEM) (FEI Quanta 200 FEG-ESEM, Hillsboro OR) and the microscope control software was used to measure the diameters of the RDX particles. Each sample was analyzed at 3.0 keV under high vacuum. The samples were coated with 10 nm of gold prior to imaging to prevent charging of the particles during SEM imaging.

## III. RESULTS AND DISCUSSION

### A. RDX particle production

**FIG. 6:**
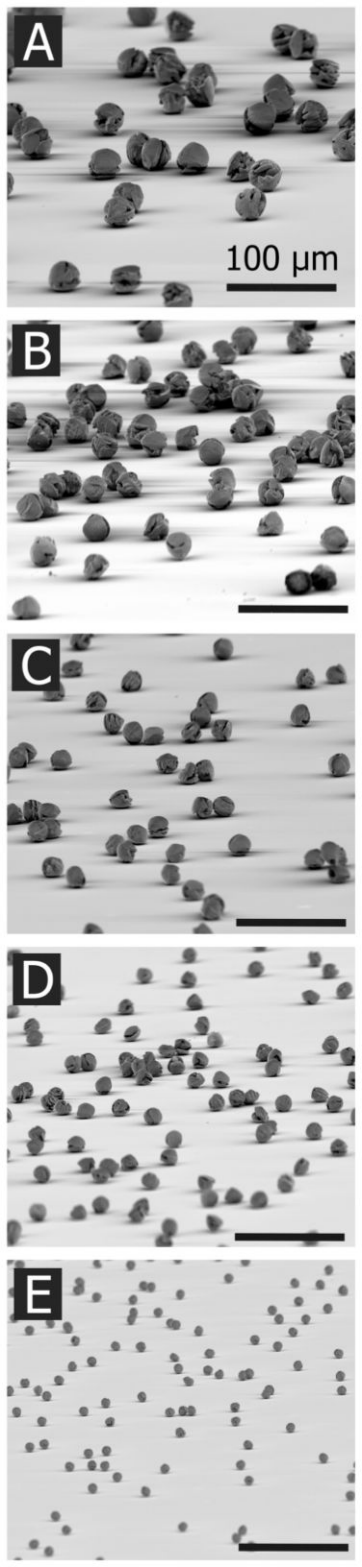
SEM images of RDX particles printed with the particle jet. Particles range from 30 μm to 9 μm in diameter. The scale bar is 100 μm in each image. Particles are printed with varying nominal concentrations of RDX in DMF: (a) 150 mg/ml, (b) 100 mg/ml, (c) 50 mg/ml, (d) 25 mg/ml, and (e) 5 mg/ml.

RDX particles were produced from RDX-DMF solutions with nominal concentrations of 150 mg/ml, 100 mg/ml, 50 mg/ml, 25 mg/ml, and 5 mg/ml. Spheroid microparticles produced by each of these solution concentrations are shown in Figure 6. The scale for each image is the same; the particle size is related to the concentration of RDX in each solution. A high degree of monodispersity is evident in each batch and particle agglomeration is not present in any of the examples shown. A droplet ejection frequency of 10 Hz completely eliminated agglomeration, however at greater dispensing frequencies the droplets exhibited a propensity to coalesce in the drying tube and form doublets or even triplets.

**FIG. 7:**
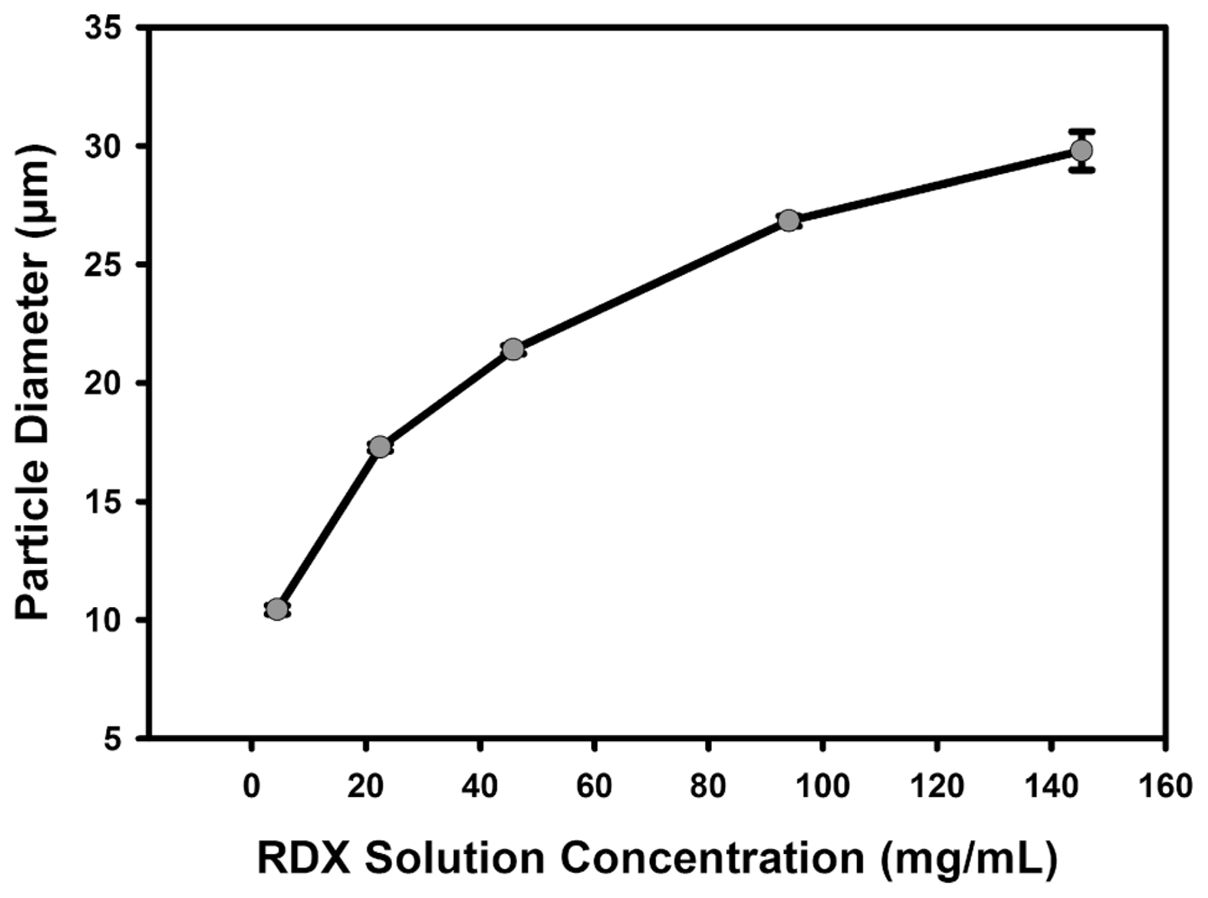
RDX particle size as a function of RDX solution concentration. Particle diameters were determined using normal-incidence SEM images and a length measurement function in software. The diameter of 30 particles at each concentration was measured. Error bars are one standard deviation and are too small to be seen in some cases.

As demonstrated in Figure 7, the resulting particle size is a function of the total amount of analyte ejected in a single droplet, which is dependent on the initial concentration of the jetting solution; D_p_ ~ C(1/3)D_d_, where D_p_ is the particle diameter, C is the concentration of analyte in solvent and D_d_ is the initial inkjet droplet diameter. In this example, the initial liquid droplet diameter was 60 μm on average which matches the behavior of the curve. Leveraging the relationship between final microparticle diameter and initial solution concentration is a key advantage to this particle production system because it enables the fabrication of a known number of quantifiable microparticles with tight control over final particle diameter.

### B. Ammonium nitrate particle production

**FIG. 8:**
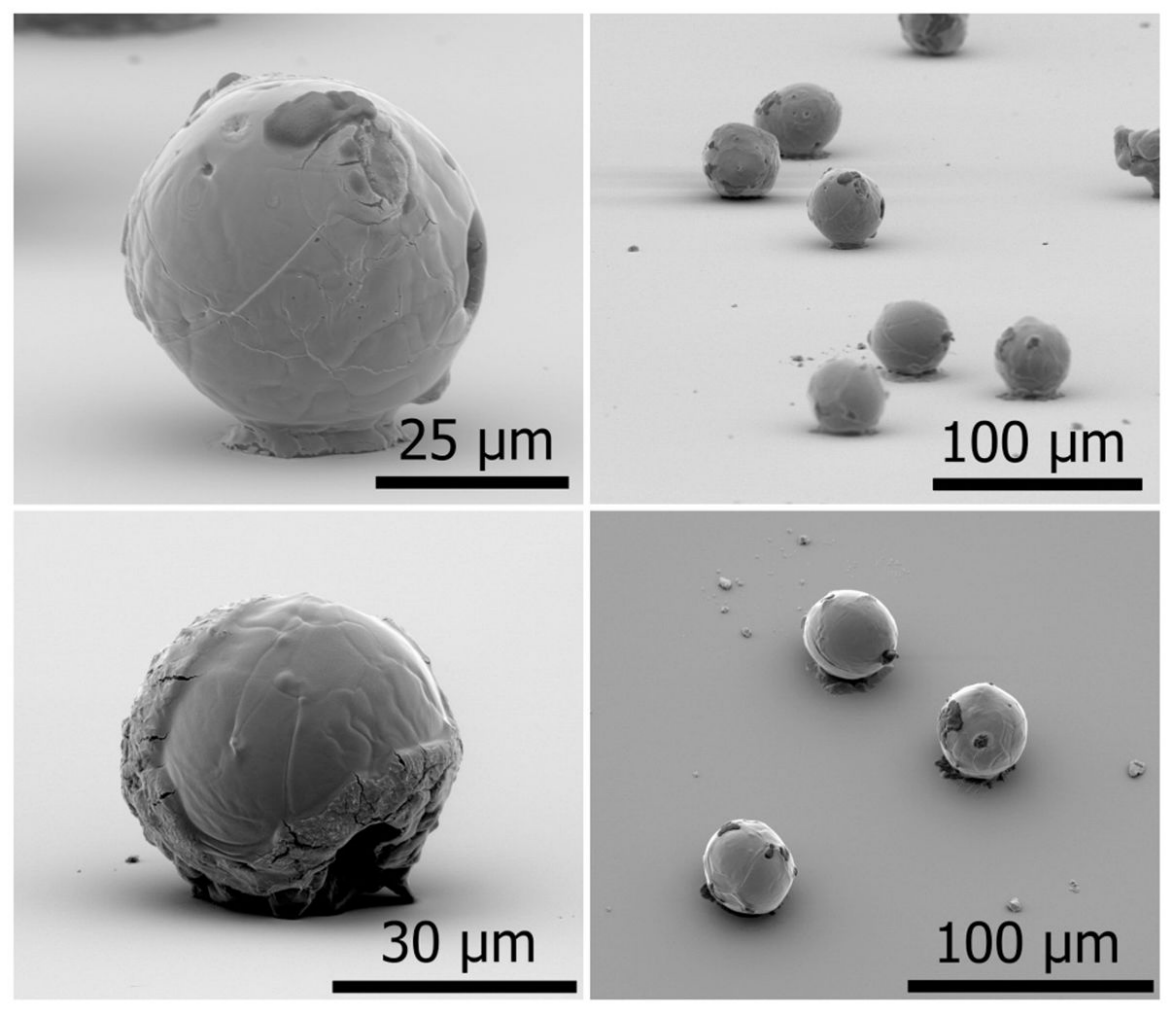
SEM images of AN particles produced by the particle jet. The particles are approximately 40 μm in diameter.

The deliquescent properties of AN made it especially challenging to generate microparticles that were completely void of water. An elevated temperature within the drying tube assisted in accelerating the evaporation of water in each droplet as it traveled down the drying tube, however, the resulting microparticles were never fully dehydrated. A collection of SEM images of AN particles, shown in Figure 8, shows that most of the particles form into a spherical shape with unusual structures and growths along the exterior surfaces. These particles are on average about 40 μm in diameter with a standard deviation of 1.2 μm. The high vacuum of the SEM surely aids in dehydrating residual water from the particles and produces images that suggest that they are completely dry. However, once the samples were in a laboratory environment at room temperature and pressure, simply breathing onto the silicon substrate that contained these hygroscopic particles would convert a fraction of them back into a liquid phase.

## IV. SUMMARY AND CONCLUSIONS

Our interest in developing trace particle test materials for security and forensics applications has motivated us to develop and test a system to produce microparticles quantitatively using a piezoelectric inkjet print system. The ability to produce custom particles of controllable size and chemistry is one critical step towards the successful evaluation and optimization of trace contraband sampling technologies.

This work presents an overview of the development and testing of a prototype particle printing device, the particle jet. An important component of the particle jet is the custom heated drying tube that facilitates semi-complete evaporation of solvent in each droplet to ultimately produce solid microparticles at the drying tube exit. The ability to push air through the tube, rather than pull air from the exit, is also a significant design feature that enables a known number of particles to be delivered to almost any surface. For example, this particle jet was used to help characterize the performance of a prototype shoe sampling system by delivering a quantified number of particles onto the surface of a shoe,^28^ and was also used to help evaluate the particle collection characteristics of a prototype particle impactor for explosives sampling.^29^

CFD modelling was performed to help visualize the flow patterns and the thermal gradients present in the drift tube. CFD also confirmed that the flow-focusing cap located at the top of the drying tube was able to redirect fluid flow downwards and prevent buoyancy forces from creating an upwards vertical flow in the central tube. Flow visualization was performed using laser light scattering on seed droplets and showed the influence of jetting frequency on droplet trajectory and spacing.

We have demonstrated the successful production of pure RDX and AN particles by the particle jet. Such particles are useful for aerodynamic studies of particle removal and collection in junction with trace chemical detection techniques such as ion mobility spectrometry. The resulting particle sizes are directly related to the analyte concentration of the printing solutions and the initial droplet diameter. Several factors were found to be important for printing pure particles, including solvent evaporation rate, analyte solubility, drying tube temperature, and the physical properties of the solvent such as viscosity and surface tension.

Future work will focus on plastic bonded explosive formulations for preparing custom explosive particle standards. Particular emphasis will be directed towards the high explosive compound C4, which contains RDX as the energetic material and polyisobutylene as a binder component. Other plastic bonded explosives such as Semtex and Detasheet are also of interest. By incorporating a binding polymer in the formulation, the lifetime of more volatile analytes may be extended because of the encapsulation properties of the polymer matrix.

Future work will also focus on the integration of an inline particle counter that counts each particle as it transits the drying tube. This addition will enable the measurement of total particle transport efficiency by comparing the number of droplets requested from the printhead to the number of particles that exit the drying tube. Additionally, the ability to characterize the adhesion of these particles on various substrates would be of great benefit to the trace explosives community and will be the topic of future activities.
